# Thyroid hormone action in adult neurogliogenic niches: the known and unknown

**DOI:** 10.3389/fendo.2024.1347802

**Published:** 2024-03-07

**Authors:** Victor Valcárcel-Hernández, Steffen Mayerl, Ana Guadaño-Ferraz, Sylvie Remaud

**Affiliations:** ^1^ Laboratory Molecular Physiology and Adaptation, CNRS UMR 7221, Department Adaptations of Life, Muséum National d’Histoire Naturelle, Paris, France; ^2^ Department of Endocrinology, Diabetes and Metabolism, University Hospital Essen, University Duisburg-Essen, Essen, Germany; ^3^ Department of Neurological Diseases and Aging, Instituto de Investigaciones Biomédicas Sols-Morreale, Consejo Superior de Investigaciones Científicas (CSIC)-Universidad Autónoma de Madrid (UAM), Madrid, Spain

**Keywords:** thyroid hormones, adult, adult neurogenesis, adult oligodendrogenesis, SVZ: subventricular zone, SGZ: subgranular zone of the hippocampus

## Abstract

Over the last decades, thyroid hormones (THs) signaling has been established as a key signaling cue for the proper maintenance of brain functions in adult mammals, including humans. One of the most fascinating roles of THs in the mature mammalian brain is their ability to regulate adult neurogliogenic processes. In this respect, THs control the generation of new neuronal and glial progenitors from neural stem cells (NSCs) as well as their final differentiation and maturation programs. In this review, we summarize current knowledge on the cellular organization of adult rodent neurogliogenic niches encompassing well-established niches in the subventricular zone (SVZ) lining the lateral ventricles, the hippocampal subgranular zone (SGZ), and the hypothalamus, but also less characterized niches in the striatum and the cerebral cortex. We then discuss critical questions regarding how THs availability is regulated in the respective niches in rodents and larger mammals as well as how modulating THs availability in those niches interferes with lineage decision and progression at the molecular, cellular, and functional levels. Based on those alterations, we explore the novel therapeutic avenues aiming at harnessing THs regulatory influences on neurogliogenic output to stimulate repair processes by influencing the generation of either new neurons (i.e. Alzheimer’s, Parkinson’s diseases), oligodendrocytes (multiple sclerosis) or both (stroke). Finally, we point out future challenges, which will shape research in this exciting field in the upcoming years.

## Introduction

Adult neurogliogenesis is a process strictly regulated in all mammals, including humans. In particular, neurogenesis occurs throughout the lifespan in specific and restricted areas of the brain. The subventricular zone (SVZ) lining the lateral ventricles (1, 2) and the subgranular zone (SGZ) in the hippocampus (3, 4) are the two main niches in which adult generation of new neural cells has been extensively described in physiological and pathological conditions. However, several other brain areas have also been identified as emerging sites of newly generated neurons and glial cells in adult mammals, although the origin of progenitors underlying this process remains controversial. Such niches include the hypothalamus (5, 6), striatum (2, 7–9), and cerebral cortex (10, 11), among others, such as the amygdala (12, 13). However, even though neurogliogenesis has already been extensively studied, mechanisms underlying the generation of new neural cells in the adult are not completely disentangled up to date.

Among the potential players controlling these processes, thyroid hormones (THs), T_3_ (3,5,3’-triiodothyronine) and T_4_ (thyroxine) arise as top candidates. The role of THs on adult neurogliogenesis has been well established in mammals, and especially in rodents, for some time now (14–16), with various aspects ranging from the control of cell proliferation (17–21), determination and differentiation (18–22), to cell death (18, 23).

However, mechanisms underlying TH-dependent neurogliogenic processes are only emerging in the two main neurogenic niches (SVZ and SGZ), but further research is needed to assess TH action in other emerging adult neurogliogenic niches. We hypothesize that a dynamic interaction between TH signaling regulators tightly modulates intracellular TH action, thus regulating neural stem cell (NSC) behavior (i.e., proliferation and neuron/glia determination) and progenitor differentiation. Cell-specific THs availability in the brain is finely tuned by (i) THs supply to cerebral tissues carried out by TH-distributor proteins such as transthyretin (TTR) (24) and ii) transmembrane TH-transporters (THTs) (25). Moreover, TH action in the brain is regulated by iii) a balance between TH-activating deiodinases (mainly type 2 deiodinase, or DIO2, in the brain, that locally converts T_4_ to T_3_) and inactivating deiodinases (mainly type 3 deiodinase or DIO3) (26). Finally, to translate the THs signal into changes in gene expression, iv) the presence of ligand (T_3_)-dependent nuclear receptors (TR) (27) such as TRα1, TRβ1 or TRβ2, is the main way for THs action.

Besides the obvious interest in understanding the molecular and cellular aspects of adult neurogliogenic processes and their interactions with THs, it is also important to note that the generation of new neural cells in the adult brain has major functional impacts on health and disease, and a better knowledge of these TH-dependent mechanisms could lead to new therapeutic avenues. Hence, in this review, we describe the features of the known, and lesser-known neurogliogenic niches in the adult rodent brain, as well as the multiple roles of THs in regulating neurogliogenic processes in both health and disease. Finally, given the lack of knowledge on several aspects addressed in this review, we point out several future challenges, trying to pinpoint the most significant knowledge gaps, that will most likely drive further research.

## Thyroid hormone action in the SVZ

SVZ-NSCs, also known as B1 cells, are astrocytic-type cells (28, 29) generated during embryonic development. After this embryonic period, “pre-B” cells enter quiescence until adulthood, where they can be reactivated (30, 31), especially following a brain injury (1, 32, 33). A fine regulation between quiescent and proliferative NSCs is required to preserve the NSC pool within the SVZ niche (30, 31, 34, 35). Elegant real-time imaging experiments demonstrated that B1 cells generate actively proliferating Transient Amplifying Progenitors (C cells or TAP) by asymmetric division (36, 37). TAPs can divide symmetrically up to three times before generating neuroblasts (A cells), characterized by their limited proliferative capacity and the expression of the specific marker doublecortin (DCX+) (37). DCX+ neuroblasts migrate towards the olfactory bulbs (OB) along a tangential migration pathway, the Rostral Migratory Stream (RMS) (38). In the OB, neuroblasts migrate radially and differentiate into distinct populations of GABAergic (expressing calbindin and calretinin) and dopaminergic (expressing tyrosine hydroxylase) interneurons (39–41) that integrate into pre-existing interneuron networks (41–43). These newly generated olfactory neurons play a role in the olfactory function of rodents, particularly in the discrimination and memorization of odors, which are crucial for the animal’s adaptation to its environment (for mating and offspring care) (43–46).

Glial cells, including astrocytes and oligodendrocyte precursors (OPCs, identifiable by the oligodendroglia lineage marker OLIG2), are also derived from a subpopulation of SVZ B1 cells (2, 47–49). OPCs derived from SVZ-NSCs migrate towards white matter tracts in proximity to the lateral ventricles (i.e., corpus callosum, striatum) where they differentiate into mature myelinating oligodendrocytes (2, 21, 50–52). Interestingly, SVZ-OPCs never produce glial cells located in OBs (53, 54). Functional studies of oligodendrocytes derived from SVZ-NSCs are limited. SVZ-OPCs are capable of successfully repairing damaged demyelinated lesions in the corpus callosum and striatum, close to the lateral ventricle (21, 51, 52). Thus, SVZ-OPCs constitute an endogenous source of myelin-enhancing oligodendrocytes in the adult mammalian brain. It is of particular relevance to stimulate this endogenous production of SVZ-OPCs in order to improve functional myelin recovery, by promoting (i) the generation of OPCs from SVZ-NSCs, (ii) the migration of these OPCs to the injury sites and (iii) the differentiation of these OPCs into mature myelinating oligodendrocytes. This question is of particular interest given that postmortem brain studies in patients who died of multiple sclerosis (MS) have shown that SVZ-derived OPCs also migrate to lesions located in the corpus callosum (50). Thus, the recruitment of newly generated OPCs in adults is conserved between humans and rodents.

**Figure 1:**
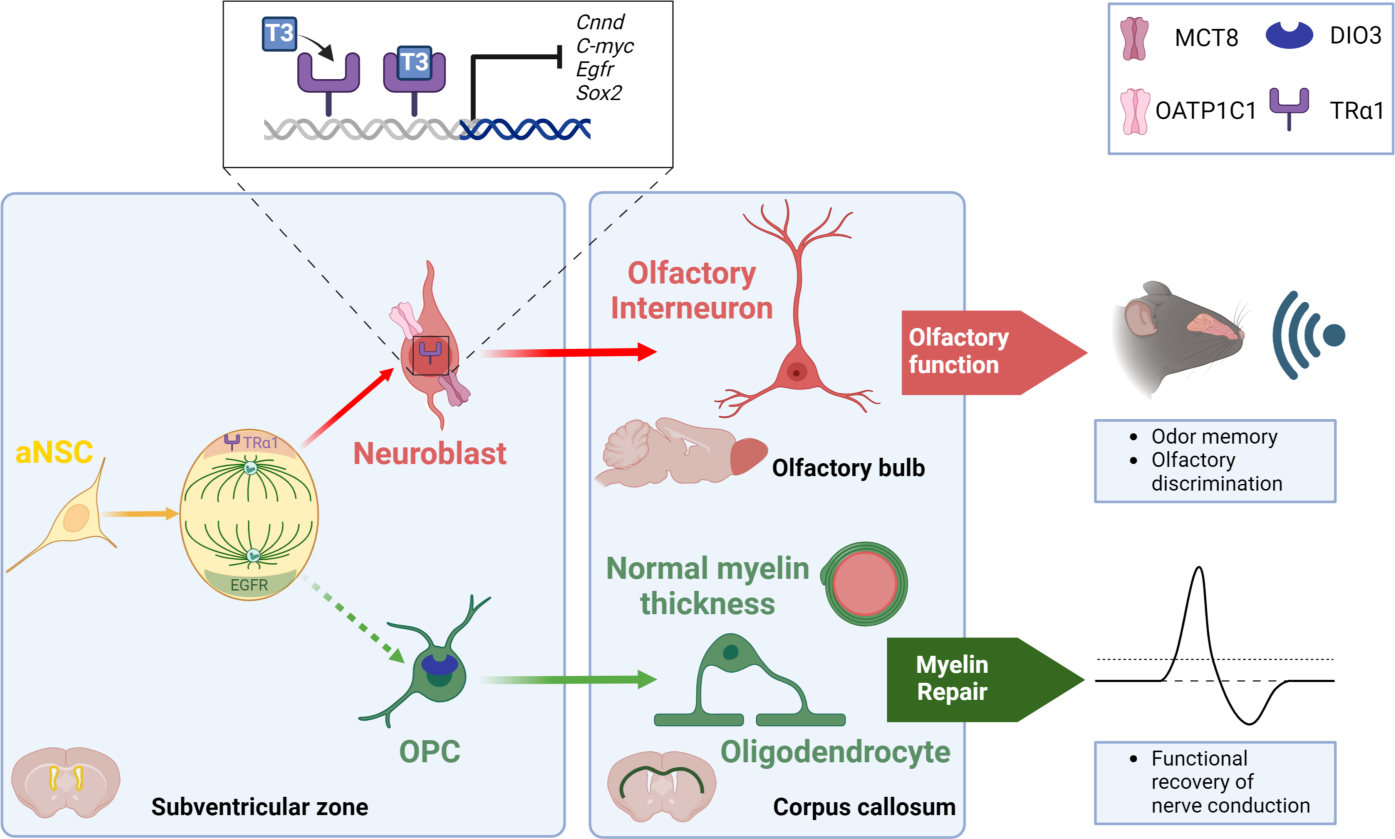
TH signaling modulates the neuronal *versus* glial fate decision within the SVZ niche of the adult mouse. During NSC division, an asymmetric segregation of a neural determination factor (TRα1) and a glial determination marker (EGFR) is observed. Moreover, T_3_ through its receptor TRα1, drives NSC commitment preferentially towards a neuroblast via downregulating various genes involved in glial determination (*Egfr*), cell cycle progression (*c-Myc*, *Cnnd1*) and NSC pluripotency (*Sox2*). Neuroblasts generate olfactory interneurons in the olfactory bulbs that participate in olfactory behavior that is strongly affected when THs action is impaired. A lack of T_3_ favors the generation of new SVZ-derived OPCs. These SVZ-OPCs can repair myelin in the corpus callosum and restore normal nerve conduction after a demyelinating insult using the cuprizone mouse model.

The role of THs in the biology of SVZ-NSCs has been investigated mainly by Remaud’s group over the past two decades in the young adult mouse (**Figure 1**). We first determined the expression pattern of several regulators of THs action (i.e, TRs, THTs, TH-distributor proteins, deiodinases) within the adult mammalian SVZ to identify the cell types that preferentially respond to THs. By immunohistochemistry, we demonstrated that only the TRα1 isoform is expressed in SVZ cells, and not TRβ (18, 55) and that TRα1 is found especially in neuroblasts (55) but not in OLIG2+ OPC (21) (**Figure 1**). Interestingly, we found that TRα1 (considered as a neuronal determinant) and EGFR (a TH-target gene involved in glial determination) are asymmetrically segregated during NSC/progenitor division, suggesting that one daughter cell inheriting TRα1 will become a neuroblast whereas EGFR+ sister cells will be determined toward an oligodendroglial fate (21) (**Figure 1**). Regarding the expression of key THTs, monocarboxylate transporter 8 (MCT8), and organic anion-transporting polypeptide 1C1 (OATP1C1) are mainly detected in committed neuronal cells (56) (**Figure 1**). Altogether, these data suggest that TH signaling is more active in SVZ-derived neuronal cells whereas oligodendroglial cells do not seem to harbor the arsenal of regulators that would allow them to respond to THs signaling. Accordingly, OLIG2+ and SOX10+ OPCs express high levels of DIO3 (**Figure 1**), the THs-inactivating enzyme that is not expressed in neuronally committed SVZ-cells (21), showing that OPCs are protected from the effects of THs not only by the expression of DIO3 but also by the absence of TRα1 expression. Moreover, Vancamp et al. (2019) reported that, although mRNAs for the TH-distributor protein *Ttr* were detected in SVZ, especially in NSCs and neuroblasts, by RTqPCR, they did not detect the TTR protein using immunohistochemistry (57). This strongly suggests that TTR-mediated THs supply could be a key factor favoring neuronal specification (57). However, the detection of *Ttr* transcripts *versus* the failure to detect TTR protein by immunohistochemistry requires further investigation to better determine the action of *Ttr* within SVZ cells. As the intracellular action of THs can be regulated at multiple levels in the targeted SVZ cells, depending on the expression of THTs, deiodinases, TH-distributor proteins or receptors, two factors that modulate the intracellular response to TH should be given a more careful consideration. First, the expression of the TRα2 isoform should be better investigated in future studies concerning the cellular and molecular responses to THs signaling, as its putative dominant-negative role (without T_3_ affinity) may counteract the intracellular action of THs. Indeed, TRα2 is highly expressed in the brain, notably in adult SVZ cells as we demonstrated by RTqPCR following FACS-dissected murine SVZs (56). Second, to gain an overview of the regulation of T_3_ availability in the various SVZ cell types, it is crucial to define the role and the expression pattern at the protein level of DIO2 that remains unexplored yet.

We established that fine tuning of intracellular T_3_ ligand availability in the different SVZ cell types governs neuron/glia fate orientation of adult murine NSCs. In particular, we demonstrated that hypothyroidism, induced by PTU (6-n-propyl-2-thiouracil) treatment, reduced the number of mitotically active cells in adult mice, by arresting NSCs and progenitors during the S phase (18). A similar phenotype was found in Trα^0/0^ mice (18), lacking all isoforms encoded by the *Thra* locus (58). Moreover, a decreased apoptosis was observed in the SVZ of PTU-induced hypothyroid mice (18, 23). A T_3_ intraperitoneal injection restored proliferation and apoptosis to levels similar to the control group, demonstrating that T_3_ is required for progenitor proliferation within the adult SVZ. Furthermore, adult PTU-induced hypothyroid mice also showed a reduction in the number of migrating neuroblasts along the RMS (18), thus negatively impacting the generation of new olfactory interneurons. In line with these findings, we showed that TH signaling acts as a neurogenic switch by promoting the commitment of NSCs and progenitors preferentially to a neuronal fate (21, 55). TRα1 overexpression using an *in vivo* non-viral gene transfer method (58, 59) increased neuroblast generation in the most dorsal part of the SVZ. We have shown that T_3_ - *via* its receptor TRα1 - promoted neuronal determination through transcriptional repression of (i) *Sox2*, a key gene involved in NSC pluripotency (55) and (ii) crucial genes involved in regulating cell cycle progression such as *Ccnd1* and *c-Myc* (18, 59). Furthermore, we also showed in 2017 that T_3_ promoted neuronal fate at the expense of oligodendroglial fate, notably through modulation of cell metabolism (15). In particular, mitochondrial respiration and fission are more active in neuronal precursors, compared to the situation in oligodendroglial precursors.

While T_3_ favors a neuronal fate, a TH-free window in contrast promoted the generation of new OPCs derived from adult murine SVZ-NSCs (15, 21). We reported *in vivo*, by immunohistochemistry, that SVZ-OPCs are protected from the T_3_-mediated pro-neurogenic effects *via* (i) a high expression of DIO3 (21) (ii) a lack of TRα1 expression and (iii) an absence of two key THTs, MCT8 and OATP1C1 (18, 21). In contrast, neuroblasts express TRα1 but not DIO3, suggesting that TH signaling is active in neuronal cells. Moreover, a transient reduction of *Dio3* expression (by *in vivo* non-viral transfection using a DNA plasmid expressing a shRNA directed against mRNA encoding *Dio3*) induced a significant decline in the number of SVZ-OPCs, illustrating the importance of this “T_3_-free window” in SVZ-oligodendrogenesis. While a transient lack of T_3_ is required for SVZ-OPC specification, T_3_ is also well known for accelerating OPC exit from the cell cycle and committing them to differentiation *via* TRα1, in cooperation with another nuclear receptor, RXRϒ (60, 61). Furthermore, we have recently shown that adult mice deficient for the TH-distributor protein TTR - exhibiting low levels of T_3_ and T_4_ in the CSF (62) - increased the generation of new SVZ-derived OPCs at the expense of the production of new neuroblasts (57). Thus, the neuron/glia balance is shifted once again in favor of oligodendrogenesis in the context of central hypothyroidism. Similarly, our latest work revealed increased SVZ-OPC production in the absence of MCT8 and OATP1C transporters using the *Mct8^-/-^, Oatp1c1^-/-^* double knockout dKO mice (56). In turn, the production of mature neuroblasts is diminished and associated with a migration defect.

What is the functional relevance of the T_3_-dependent regulation of the neuron-glia balance? As mentioned previously, T_3_ preferentially drives NSC fate towards neuroblasts. In various pharmacological and genetic backgrounds, that reflect a central hypothyroidism, we have shown that reduced SVZ-neurogenesis is associated with impaired olfactory behavior in adult mice, and in particular with a reduced short-term olfactory memory (56) (**Figure 1**). We also examined the functionality of the “T_3_-free window” in a well-established model of demyelination using cuprizone, a gliotoxin that induces death of mature oligodendrocytes (63) predominantly distributed in the corpus callosum for a six-week cuprizone treatment period (21). Transient hypothyroidism was then applied to mice during the demyelination phase, a phase during which new SVZ-OPCs are reported to proliferate, thus promoting myelin repair (64). In that context, we have shown that these mice efficiently repaired demyelinating lesions in the corpus callosum, especially just above the ventricles. Indeed, waves of remyelination originating in the dorsal part of the SVZ are puzzlingly observed. Unexpectedly, quantification of myelin thickness by electron microscopy revealed that myelin sheath at remyelination sites is of normal thickness around the axons of the corpus callosum (21). Furthermore, electrophysiological experiments were performed by measuring compound action potentials (CAPs) in coronal corpus callosum slices one week after endogenous remyelination (64). We highlighted that such hypothyroidism, applied during demyelination, enabled a functional recovery of nerve conduction (21) (**Figure 1**). Similarly, TTR null mice also displayed an increase in oligodendrogenesis during development (65) as well as a thicker myelin sheath in the corpus callosum following cuprizone withdrawal (66). Thus, SVZ-derived OPCs constitute an endogenous source of glial progenitors capable of functionally repairing a demyelinated lesion localized in the corpus callosum, close to the lateral ventricles as it has been also demonstrated by other works (51, 52). However, how a TH-deficient environment contributes to restoring a myelin sheath of normal thickness is still an unresolved question.

Our findings on TH action on SVZ-NSCs led to TH signaling being considered as a potential key signal for stem cell-based regenerative medicine (67). The regenerative potential of THs has been well documented in fish, maintaining a large adult pool of NSCs (68, 69). In contrast, in mammals, which exhibit a neonatal THs peak [for review, see (70)], CNS regenerative capacities are drastically diminished after this THs peak. The adult SVZ niche, which remains sensitive to THs throughout life, provides a potential source of neural cells that can be mobilized in pathophysiological conditions requiring the supply of new neurons (as seen in Alzheimer’s) or oligodendroglial cells (as observed in MS), or both (as in the case of stroke). Here, we focus on the contribution of THs in repairing demyelinating lesions that are characteristic of MS, a chronic inflammatory disease that affects the entire CNS (brain and spinal cord). It is the first cause of motor disability in young adults. In addition, the worldwide incidence of this disease has been unexpectedly increasing over the last twenty years (71) and therefore represents a major public health issue. MS is associated with multiple demyelinating lesions (plaques), inflammatory cells, loss of oligodendrocytes, and decreased axon density (72). A burning question is to understand how to mobilize the cell type that favor remyelination. OPCs, which account for 5% to 8% of adult CNS cells, and the myelin-forming oligodendrocytes derived from them, are obvious targets for promoting myelin repair [for review, see (73)]. One approach to boosting myelin recovery is to enhance the pool of oligodendrocyte progenitors. Two endogenous sources can be mobilized for MS patients: (i) newly-generated adult OPCs from NSCs, as discussed above, and (ii) parenchyma-resident OPCs (pOPCs), generated during development and which persist in the adult brain. Thus, the SVZ represents an attractive endogenous source of OPCs. Postmortem examination of brains from individuals diagnosed with MS revealed efficient recruitment of SVZ-derived OPCs to sites of injury in the corpus callosum (50). The rodent model of cuprizone-induced demyelination, as mentioned above, has been used to assess the effect of THs on remyelination. Franco et al. (2008) showed that T_3_ injections during the recovery/remyelination period - following 2 weeks of cuprizone-based demyelination - induced an increase in OLIG2+ oligodendroglial cells exiting the cell cycle in the SVZ along with increased detection of TRα1 and TRβ in SVZ cells by immunohistochemistry. An increased number of mature oligodendrocytes expressing hallmark markers (O4, MBP, PLP, CC1) was observed in the corpus callosum (74). Similarly, an MRI analysis also showed that T_3_ injections in mice, during the recovery weeks after cuprizone treatment, enhanced remyelination in the corpus callosum (75). In these two studies, however, the origin of the remyelinating cells (SVZ-derived OPCs or pOPCs) was not clearly stated. Since then, work several groups including ours (21, 51, 52), has shown - using cell tracing experiments - that adult murine SVZ OPCs were able to migrate to myelin damage induced in the corpus callosum and then differentiate into mature myelinating oligodendrocytes. Our protocol - based on a transient hypothyroidism window applied during the demyelination phase followed by T_4_/T_3_ pulses during recovery - allows to restore a myelin sheath of similar thickness to that of the euthyroid control group. In contrast, pOPCs did not respond to this transient hypothyroidism, unlike newly generated OPCs, showing that these two OPC subpopulations respond differentially to THs signaling. The underlying challenge would be deciphering the molecular mechanisms that regulate the differential response to THs of SVZ OPCs (complete remyelination) and pOPCs (incomplete remyelination), in order to better understand the mechanisms responsible for functional myelin repair.

### Future challenges:

- What is the function of some regulators (i.e., DIO2, TTR, TRα2) in the physiology of adult SVZ-NSCs? In particular, the role of TTR as a TH-distributor protein in the CSF and/or a function independent of THs should be better deciphered. Furthermore, the function of the TRα2 isoform would deserve more in-depth consideration since TRα2 is highly expressed in adult SVZ cells and may act as a dominant-negative regulator (with no affinity for T_3_) countering intracellular THs effects.
- How do THs differentially regulate the response of SVZ-derived OPCs *versus* resident OPCs following a demyelinating lesion?
- Non-genomic effects, that do not require an interaction between T_3_ and its TR, are not yet known on NSC proliferation and determination within the SVZ niche and should be assessed in further studies

## Thyroid hormone action in the SGZ

Adult hippocampal neurogenesis is a highly orchestrated process that continuously generates new granule cell neurons throughout life. To this end, the SGZ of the dentate gyrus harbors radial glia-like NSCs that express markers like GFAP or Nestin as shown in rodents (14, 76, 77). Though they can also differentiate into astrocytes, NSCs mainly divide asymmetrically thereby giving rise to rapidly proliferating TAPs that are also referred to as type 2 progenitors (76, 78). Based on their expression profile, this population is further sub-divided into Nestin positive, type 2a progenitors, which then develop into DCX positive, type 2b progenitors (14, 76). Subsequently, these cells form into neuroblasts, which exhibit a reduced proliferative capacity, eventually exit from the cell cycle, and differentiate into immature granule cell neurons (76). Both neuroblasts and immature neurons continue to express DCX but can be distinguished by the presence of the calcium-binding protein calretinin in immature neurons (79). The sequential progression through these distinct stages is a rapid process and in mice, new immature neurons are derived from NSCs within three days (79). The final steps encompassing short-distance migration, maturation, and functional integration of new neurons into the existing granule cell network take 4-6 weeks in rodents (76). This continuous generation of new neurons significantly contributes to the high plasticity of the adult hippocampus. Several studies have demonstrated a critical role for adult hippocampal neurogenesis in cognitive flexibility and more specifically in learning and memory processes, in emotional regulation, anxiety, and spatial navigation (78, 80, 81). Although much better characterized in the rodent brain, observations in humans that demonstrated the presence of DCX+ cells and detected new neurons in the hippocampus strongly argue for the existence of adult hippocampal neurogenesis also in humans (77, 78).

**Figure 2:**
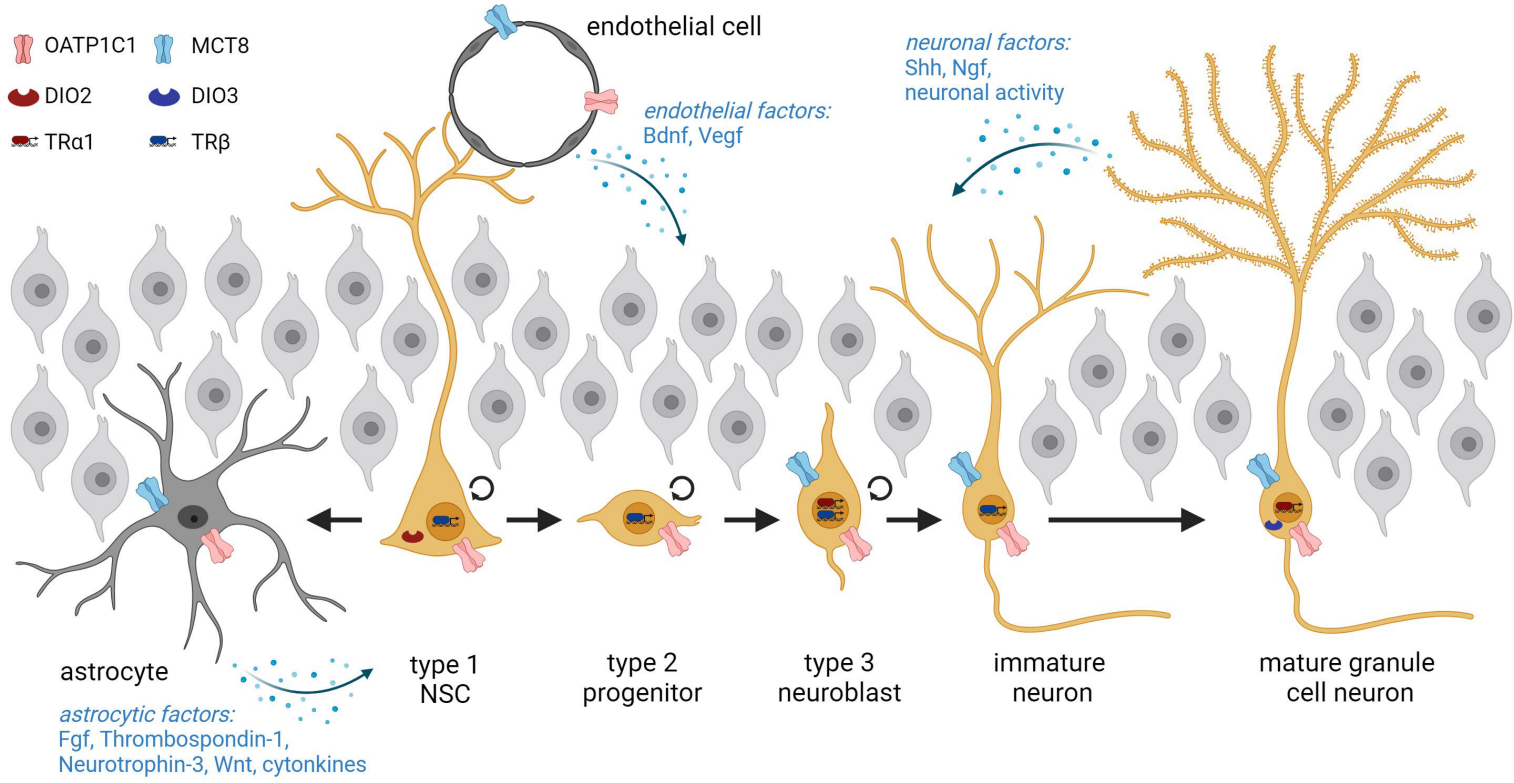
Overview of adult hippocampal neurogenesis starting from NSCs in the subgranular zone and progression through the different stages. NSCs are in intimate contact with the endothelium and can either generate type 2 progenitors or differentiate terminally into astrocytes. The cellular expression of THs transporters and deiodinases within adult hippocampal lineage cells is depicted. Neurogenesis is further regulated by extrinsic factors derived from astrocytes, endothelial cells, and neurons. Graphic was created with Biorender.

THs constitute an important extrinsic signaling cue for adult hippocampal neurogenesis and components of the TH signaling pathway were identified at all stages of the program in mice. Transcript analyses on isolated neurogenic populations revealed the expression of the THs transporting amino acid transporters *Lat1* and *Lat2* in SGZ-NSCs and type 2 progenitors (19) (**Figure 2**). *In vitro* and *in vivo* studies highlighted the presence of TRβ isoforms in Nestin positive as well as in proliferating, BrdU positive hippocampal progenitors whereas TRα1 was mainly detected in DCX+ neuroblasts and granule cell neurons (82, 83). Likewise, we demonstrated that the latter two populations are equipped with the highly specific THT MCT8 while mature granule neurons further express MCT10, LAT2, and DIO3 (19). *Oatp1c1* promoter activity was detected in subsets of all progenitor and neuronal populations though the causes and consequences of this heterogeneity remain to be investigated (84). Together, the expression patterns of TH signaling components suggest that progenitor and mature neuronal populations within the adult hippocampal neurogenic lineage possess the ability to directly sense and integrate the TH signal.

A wealth of experimental studies has addressed the consequences of manipulated THs availability on neurogenesis in the adult SGZ and demonstrated THs-induced effects predominantly during the later stages of the neurogenic program (14, 19, 20, 82, 83, 85, 86). Adult-onset hypothyroidism led to decreased precursor cell survival and a reduced number of DCX positive progenitor cells in rodents (20, 22, 83, 85, 87, 88). These observations, however, were made in animals in which experimental interventions caused a strong decrease in circulating THs levels. Low-dose PTU treatment induced adult-onset hypothyroxinemia (subclinical hypothyroidism) in rats that did not result in similar alterations in SGZ neurogenesis indicating that most likely the extent of THs insufficiency is critical (89). T_3_-induced, adult-onset hyperthyroidism conversely accelerated progenitor differentiation in mice (22). Along this line, the level of TRα1 correlates inversely with the total number of DCX positive cells. TRα1 null (TRα1^-/-^) mice harbor elevated numbers of DCX positive cells, while the same cell population is reduced in TRα2^-/-^ mice, which overexpress TRα1 6-fold as both isoforms are derived from the same gene through alternative splicing (82). Rather than to the absence of the non-TH-binding TRα2 isoform in the latter model, the reduction in DCX positive cells has been attributed to the increased concentration of TRα1 that in light of limited cellular T_3_ availability generates a condition comparable to local hypothyroidism. Together, these findings point to a negative impact of the TRα1 aporeceptor on lineage progression. Our studies further showed that absence of MCT8 either globally or specifically in the adult neurogenic lineages using a tamoxifen-inducible form of Cre recombinase expressed under the Nestin promoter (Nestin-CreERT2) diminished the differentiation to immature neurons and the formation of new granule cell neurons (19). Mechanistically, this has been linked to a delayed cell cycle exit through decreased expression of the cell cycle inhibitor p27Kip1 in DCX positive cells *in vivo*. Similarly, the global absence of OATP1C1 impaired progenitor differentiation and program progression, but whether this reflects a lineage-intrinsic function remains elusive (84). This is but one example for the future challenge to identify the lineage-autonomous impacts of TH signaling components. Likewise, the question as to which pathways mediate TH effects on hippocampal neurogenesis *in vivo* awaits further investigation.

In contrast to the well-established action in later stages, the potential impact of THs on early progenitor populations in the adult SGZ remains controversial. While adult-onset hypothyroidism in rats exposed to PTU or methimazole (MMI) did not result in altered BrdU incorporation into cycling progenitors, their number was reduced in adult-onset thyroidectomized rats, an effect that was rescued by THs administration to the drinking water (20, 83, 85). Moreover, the global absence of TRβ in mice stimulated progenitor proliferation and BrdU incorporation in the SGZ indicating a regulatory function of THs/TRβ in NSC activation and progenitor proliferation (90). It has been suggested that unliganded TRβ exerts a repressive function on NSC turn-over, which would be lifted upon T_3_ binding (67). This scenario, however, requires the uptake of THs into SVZ-NSCs through a yet unidentified pathway. Recent hippocampal single cell and single nuclei RNA sequencing studies advanced this idea and highlighted the presence of *Oatp1c1* transcripts in NSCs in line with *in vivo Oatp1c1* promoter activity studies (84, 91–93). Interestingly, these sequencing studies also detected *Dio2* transcript expression in murine SGZ-NSCs. Although further work is still needed, it is a fascinating idea that NSCs are equipped with the machinery to take up T_4_ and generate T_3_ in a cell-autonomous manner, as has been proposed to take place in radial glial cells in the prenatal human cerebral cortex (94). At the same time, super-resolution microscopy revealed that hippocampal NSC processes contact intimately the blood-brain barrier (BBB) and are capable of directly accessing blood-born substances (95). In this context, NSCs were shown to take up BBB-impermeable substances in an otherwise unimpeded BBB environment stressing how privileged this access is. It is thus tempting to hypothesize that NSCs directly take up T_4_ from the circulation via up-regulation of OATP1C1 in the activated state, and convert it locally to T_3_, which in turn stimulates NSC turn-over. The T_4_-dependency of such a scenario may explain the apparent lack of effect in T_3_-induced hyperthyroidism (22). Moreover, due to their slow cell cycle kinetics and high degree of quiescent NSCs, the consequences of altered THs signaling on NSC activation may be mild and may only become obvious after a longer time interval than is usually assessed in adult-onset hypothyroid models (96). Such a regulatory influence on NSC activation may further explain the preservation of NSC numbers with age as seen in *Mct8* knockout (KO) and *Mct8/Oatp1c1* dKO mice potentially associated with their reduced T_4_ serum levels and different degrees of central THs deficit (84, 97). Though acting through a different pathway, THs feature a similar function on the regulation of NSC maintenance and activity in the SVZ (16).

Importantly, modulating the thyroidal state in adult rodents also affects hippocampus-related behaviors that depend on proper SGZ neurogenesis such as spatial memory or the regulation of anxiety and mood (80, 81). Adult-onset hyperthyroidism compromised learning and memory function and increased anxiety in rats and mice (98, 99). Likewise, hypothyroidism in adult rodents either by thyroidectomy or administration of PTU or MMI as well as a low iodine diet resulted in an anxiety-depression-like state as well as impaired learning and spatial memory performance (20, 100–103). Why adult-onset hypo- and hyperthyroidism culminate in similar behavior impairments remains elusive. Though the underlying molecular pathways certainly differ, these observations suggest a gatekeeper function of balanced THs signaling for hippocampal functions. Anxiety-depression-like behaviors were further observed in TRα1 mutant mice, in which THs binding affinity is reduced 10-fold, and in global *Mct8* KO mice (84, 104). Whether the behavioral changes in the latter genetically modified models are solely the consequence of a perturbed adult hippocampal neurogenic program or if developmental alterations contribute to it, remains elusive. Definite answers require the detailed analysis of inducible KO models that lack TH signaling components specifically in adult NSCs and, consequently, their progeny.

In addition to these lineage-intrinsic mechanisms, adult hippocampal neurogenesis is under the control of non-cell-autonomous signaling cues derived from the stem cell niche. Within the mammalian SGZ, the niche encompasses astrocytes, BBB endothelial cells, microglia, and granule cell neurons as prominent cell types (105). Several lines of evidence suggest that an altered astrocytic response can mediate parts of the effects of modulated THs levels on the adult neurogenic program. First, astrocytes express DIO2 (106) and are thus central in regulating brain T_3_ availability and action (106–108). Second, astrocyte-specific deletion of *Dio2* in mice results in an increased anxiety-depression-like phenotype and thus in a pathological condition that has been associated with impaired hippocampal neurogenesis before (109–111). Though BrdU incorporation was not affected in this mouse model, a detailed characterization of the neurogenic stages is still pending. Third, astrocytes secrete TH-regulated factors like Fibroblast growth factors, Thrombospondin-1, Neurotrophin-3, or WNT ligands that in turn influence the neurogenic program (14, 112, 113) (**Figure 2**). RNA sequencing studies on cultured astrocytes exposed to T_3_ revealed alterations in WNT signaling components with a direct up-regulation of *Wnt7a*, which is a known regulator of SGZ progenitor proliferation, differentiation, and dendritic arborization (113, 114). Astrocytes and microglia further release cytokines that are involved in progenitor proliferation and survival (78). Similarly, TH-regulated signals derived from the vasculature like BDNF or VEGF, neuronal factors such as SHH and NGF, and neuronal activity converge on the progression of hippocampal precursors through the neurogenic program (17, 112, 115–117) (**Figure 2**). Yet, we have just begun to decipher the non-cell-autonomous mechanisms in niche cells by which THs influence progression through the adult hippocampal program. Inducible and conditional KO approaches will certainly broaden this exciting field of research in the future.

Whether adult hippocampal neurogenesis occurs in humans is still under debate (105, 118, 119). A growing number of studies demonstrates the presence of DCX+ cells in the human SGZ though the question as to the comparability of the molecular signature between rodent and human neuroblast markers has been raised following the detection of DCX in neurons (120). Single cell and single nuclear sequencing studies on human hippocampal tissue are still sparse and often do not depict NSCs and neurogenic populations while a neurogenic trajectory can be clearly delineated in the macaque brain (120–123). Until the existence of adult human hippocampal neurogenesis is unequivocally demonstrated and a marker repertoire established, it is very difficult to pinpoint both the expression of THs signaling components and TH-regulatory effects on adult human hippocampal neurogenesis.

Despite these difficulties, a wealth of clinical data evidences a link between an altered thyroidal state and affected hippocampus-related cognitive functions in humans. Hypothyroidism in adulthood results in anxiety, depression, specific spatial and associative memory impairments, and dementia as well as a decreased hippocampal volume (14, 16, 109, 124–126). T_4_ supplementation is able to improve cognitive perturbations in sub-clinically, but not overt hypothyroid subjects in tests addressing hippocampal functions (126). Interestingly, adult-onset hyperthyroidism culminates in cognitive impairments, anxiety, and depression (14, 109, 127). In sum, the pathological alterations seen in humans align with changes in experimental animals with abnormal thyroidal states that have been linked to impairments in adult hippocampal neurogenesis.

### Future challenges:

- What are the lineage-autonomous effects of THs within the adult hippocampal neurogenic program? In particular, is there a role for THs in the regulation of NSC physiology?
- Do TH action in stem cell niche cells contribute to regulating adult hippocampal neurogenesis in a non-cell-autonomous manner?
- Do THs contribute to the regulation of adult hippocampal neurogenesis in humans?

## Thyroid hormone action outside canonical neurogenic niches

### TH and neurogliogenesis in the hypothalamus

Neurogenesis in the hypothalamus was first reported two decades ago (6, 128) and has been stated to occur in different areas within the hypothalamus with important functions in metabolism, feeding, and sexual behavior. First, it has been demonstrated to occur in ependymal cells lining the third ventricle and more widely also in tanycytes (129). Moreover, hypothalamic DCX+ neuroblasts have been described in rodents, sheep, and humans, although with slight variations in distribution patterns, but mainly in the arcuate nucleus (129–133) with progenitor cells also producing a variable percentage of astrocytes (131). As for adult hypothalamic oligodendrogenesis, its existence had not been proven in rodents until very recently when it was unequivocally shown that the median eminence of the hypothalamus can give rise to new OPCs (5, 134).

Implications of hypothalamic neurogenesis are wide, and rank from repair after tissular lesions to influence on sexual behavior and weight control (135). As for adult hypothalamic oligodendrogenesis, in rodents it is associated with the regulation of energy balance and hypothalamic leptin sensitivity (5).

Interestingly, the hypothalamus is known to be a strongly THs regulated brain area in mammals. From early studies reporting the expression of DIO2 mostly in the median eminence (136), evidence has accumulated reporting the expression of the different TH regulators in the rodent brain. In particular, deiodinases (107, 137, 138) and THTs (139) are detected in high abundance in ependymal cells lining the third ventricle and in tanycytes, with the latter expressing DIO2, OATP1C1, and MCT8, suggesting that TH action is important in this cell type (107, 138, 139). Moreover, this strong expression of various TH regulators, including DIO2 and DIO3, THTs such as MCT8, and receptors TRα and TRβ has also been reported in the human hypothalamus (140, 141).

**Figure 3:**
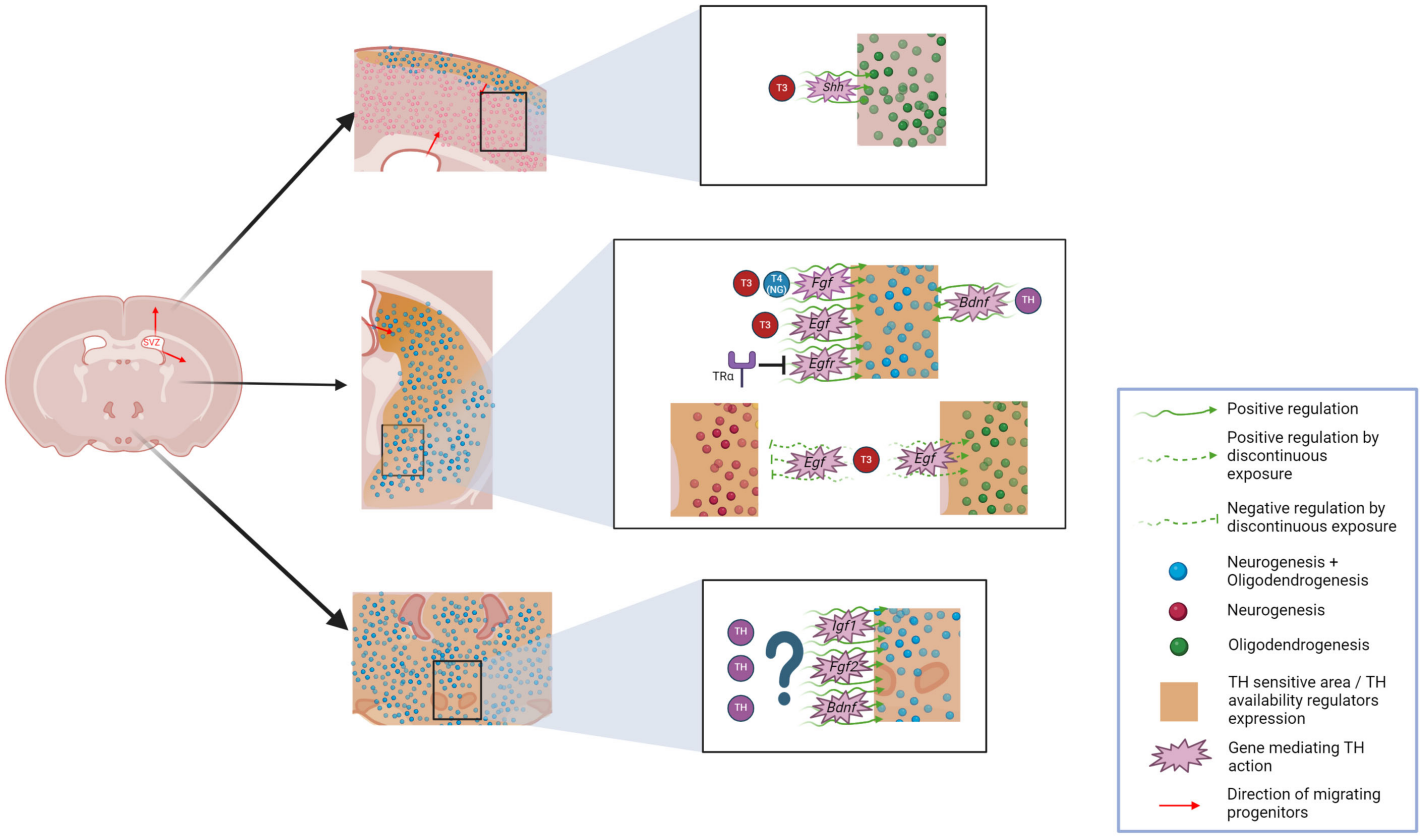
Overview of potential THs-effects on adult neurogliogenesis occurring on emerging niches including the hypothalamus, striatum and cerebral cortex. Generation of neurons and glia in the adult mammal brain is depicted for these niches and THs-influenced areas are highlighted. Potential genes mediating this THs-effect are also included. Graphic was created with Biorender.

Neurogenesis in the adult rodent hypothalamus has been also demonstrated to be controlled by growth factors (GFs) such as FGF2 (129), BDNF (130), and in a wider fashion by insulin-like growth factor 1 (IGF1) (**Figure 3**). While the first GF acts mostly on ependymal cells, the latter can act also on tanycytes (142) which could be considered a mostly IGF1-responsive cell population. Interestingly, apart from the aforementioned link between FGF2, BDNF, and THs, IGF1 has also been reported to have a strong interplay with THs (143) (**Figure 3**). Indeed, depletion of TH-availability regulators such as DIO3 or MCT8 and OATP1C1 induces increased or reduced IGF1 dynamics, respectively, in different tissues including the brain (97, 144). Altogether, GFs influence further supports a potential effect of THs on hypothalamic neurogenesis. Furthermore, mechanisms underlying hypothalamic oligodendrogenesis have not been described until very recently. However, genes consistent with the genomic footprint of hypothalamic OPCs include *MYC* and genes involved in the notch pathway (145). Interestingly, both are known to be TH-regulated within the adult mammalian SVZ and the postnatal cerebral cortex, respectively (18, 113), suggesting that THs may regulate hypothalamic oligodendrogenesis in a similar way to the situation in the SVZ and other parts of the brain.

Upon considering the important hypothalamic functions regarding regulation of energy balance and metabolism, as well as sexual behavior, and their potential association with THs, understanding the link between THs and hypothalamic neurogliogenesis could be crucial to better understand several pathophysiological processes. Regarding weight regulation, mediated by hypothalamic neurogenesis, a better understanding of THs’ role in modulating hypothalamic neurogliogenesis may be a significant advance in the prevention of pathological weight gain or loss and metabolic dysfunction usually associated with thyroid-related disorders (146–148). Furthermore, considering the common clinical implications of hypothalamic demyelination in fatigue and weight dysregulation associated to MS, enhancing hypothalamic remyelination in this region could also improve the quality of life of MS patients (149).

With all this in mind, both in physiological and pathophysiological contexts, THs appear as actors to be studied in depth in the hypothalamus, not only with the aim of understanding their role but also to potentially manipulate them to impact neurogliogenesis and thus lead to therapeutic outcomes.

### Future challenges:

- What is the ontogeny of oligodendroglial progenitors in the median eminence?
- Does hypothyroidism and/or other changes in TH availability alter neurogliogenesis in the hypothalamus?
- Would it be possible to manipulate TH availability or TH downstream effectors expression at the cellular level to regulate neurogliogenesis as a therapeutic approach for different pathological conditions, while avoiding the side effects derived from excessive TH signaling?

### TH and neurogliogenesis in the striatum

Although neurogenesis in the adult basal ganglia, particularly in the striatum, was already suspected towards the end of the past century, and later stated in rodents (150, 151) it was not until, 2014 that Ernst and colleagues demonstrated it in humans (7), by using C14 retro-tracing. In the meantime, adult oligodendrogenesis in this area was also demonstrated (2, 50, 152).

Neurogenic potential in the striatum tends to be overlooked, however, high DCX expression levels in the striatum have been reported both by transcriptomic and protein analysis, as shown previously for other neurogenic niches such as the hippocampus (153, 154). Neural progenitors in the striatum originate mainly in the SVZ. SVZ cells exhibit great heterogeneity and are able to migrate to several areas, including the classical migration to the olfactory bulbs, but also to the cerebral cortex, amygdala, or the striatum (8, 155). Moreover, studies in rodents have demonstrated active adult gliogenesis in the striatum, independent from SVZ input (156). Cell fate determination of neural progenitors is dependent on a plethora of factors and has been proven to be modulable in several ways (130, 151, 156), however, THs have never been proposed as one of those ways for striatal neuro-gliogenesis.

Adult striatal neurogliogenesis is not only interesting from a cellular ontology point of view but also has potential translatable implications to pathology. Of particular relevance are two pathological contexts, ischemic/stroke insults and neurodegenerative diseases. First, brain ischemia damages CNS tissues and in response, induces neurogenesis, oligodendrogenesis, and angiogenesis together with astrogliosis (157) to enhance brain repair. After an ischemic insult, the SVZ has been demonstrated to produce neuronal and oligodendroglial precursor cells that migrate into the striatal tissue using blood vessels as guidance and scaffold (158–161) in a process modulable by GFs (162, 163). These newly generated neurons have the ability to fully integrate and generate functional synaptic contacts within striatal neuronal networks (164) although this mechanism has been observed to be weaker in primates than in rodents (165). As for post-ischemic striatal oligodendrogenesis, it involves local pOPCs and SVZ progenitors that are recruited to the lesioned area, where they are able to exert neuroprotective effects and effectively generate myelin, although myelin has been reported thinner when produced by pOPCs (51, 161).

As for neurodegenerative diseases, Huntington’s disease (HD) outcome is strongly linked to the extent of striatal neurogenesis, as reviewed by Jurkowsky and colleagues (135). First reports in HD subjects reported reduced, if not depleted neurogenesis (7) However, later reports in animal models of the disease, particularly in rodents, and in human HD samples have reported increased adult striatal neurogenesis in concurrency of HD (166–168). This process has commonly been linked to an increase in the arrival of SVZ-derived progenitors to the striatum (168). However, it has also been reported to be closely related with local striatal astrocytes gaining neurogenic function (167), with neurogenesis from both origins potentially acting as a recovery mechanism for the cell loss associated to the disease, even in humans.

It is worth noting that the striatum is also strongly influenced by THs. Expression of some TH-availability regulators was first reported in the striatum more than 40 years ago, with evidence of striatal DIO2 and DIO3 activity among the highest in the rodent brain (169). However, we had to wait until the present century to unravel the local expression of THTs, such as MCT8 and OATP1C1, both in mice, non-human primates, and humans, with a particularly high expression of OATP1C1 (170–172). Indeed, our recent work described the expression of both transporters in striatal motor neuron circuitry, as well as in pericytes in the primate striatum, implying the importance of THs in this area (172). Moreover, in rats, thyroidectomy and subsequent depletion of THs in the striatal area have been demonstrated to induce a number of transcriptomic changes (173). After THs depletion, T_3_ administration to hypothyroid rats proved that THs modulate several genetic pathways, including genes involved in neurogliogenesis, such as *Egr1*, involved in maintaining NSC proliferation or *Klf9*, crucial to oligodendroglial cells differentiation (174, 175).

However, cellular mechanisms underlying THs regulation of neurogliogenesis within the striatum are not established yet. This regulation, even if it has not yet been established, likely occurs in relation to the GABAergic system. It has been demonstrated that a majority of the generated neurons in the adult striatum are GABAergic interneurons (7, 150, 176). Control of GABAergic system homeostasis is one of the hallmarks of THs action, and THs depletion or deficient signaling, mainly exerted through TRα have been proven to reduce the number of GABAergic cells (104, 177, 178). This reinforces the idea of an important effect for THs in striatal neurogenesis.

Although the molecular mechanism for this potential control has not been reported, among the different substances able to control adult striatal neurogenesis, GFs have been demonstrated to be crucial and stand out as potential THs downstream effectors. EGF, FGF and BDNF have been reported to increase the generation of new cells to different extents, including neurogenesis and oligodendrogenesis, both in health and disease (151, 152, 162, 179–182) (**Figure 3**).

Interestingly, all those molecules have been demonstrated to be dependent on TH signaling, with effects reported from both T_3_ and T_4_ (55, 116, 183–185). These data back up the hypothesis that local and temporal control of TH availability is crucial to neurogliogenesis, and so are the components involved in this control. It is interesting to observe that, given the importance of both GFs to striatal neurogliogenesis, they have been reported to be regulated also by different controllers of TH availability, such as deiodinases and THTs, whose depletion is able to change the expression of both GFs, through TH signaling (97, 144).

Once the putative effect of THs in adult striatal neurogliogenesis has been defined, it is necessary to understand its potential in a pathophysiological context. Aside from the aforementioned potential GFs-mediated influence in pathologies such as ischemia and HD, that remains speculative, there are reports in the literature of THs’ influence in the striatum affecting pathophysiological contexts. Interestingly, an increase in the vulnerability of striatal medium spiny neurons to HD has also been reported in deficient TH signaling, which would point to an important effect of THs in this process (186).

Although knowledge of THs influence on striatal neurogliogenesis is scarce, there is growing evidence that suggests that THs should not be overlooked as actors in the process. Further studies should be pursued in the matter, as being able to control the process of neurogliogenesis using THs regulation may be a great step forward in the managing of different pathological conditions.

### Future challenges:

- What is the ontogeny of the local progenitors present in the striatum that do not belong to SVZ derived cells?
- Does hypothyroidism and/or other changes in TH availability alter neurogliogenesis in the striatum?
- Would it be possible to manipulate striatal TH signaling as a therapeutic approach for different pathological conditions?
- Would THs manipulation induce deleterious side-effects that overcome the potential positive effects of regulating striatal neurogliogenesis?

### TH and neurogliogenesis in the cerebral cortex

For some time now, growing evidence highlights that not only cortical oligodendrogenesis but also neurogenesis occurs after embryogenesis, notably through adulthood in the mammalian brain, with important implications in disease states. Adult cortical oligodendrogenesis has been observed in rodents and humans, in physiological and pathophysiological states (2, 8, 50, 187).

In normal conditions, adult cortical neurogenesis, particularly in humans, remains controversial. After years of a fixed paradigm of non-existent cortical neurogenesis, in, 1999, Gould and colleagues claimed the discovery of newly generated neurons in the macaque cortex, originating from ectopic migration of SVZ-derived progenitors (188). However, this discovery was rejected shortly afterward by Kornack and colleagues (189), although other groups obtained similar data using different monkey species (12). Gould’s data were confirmed also by the successful generation of neurospheres from human cortical progenitor cells (190). However, C14 studies both in healthy samples and in cortical stroke-affected individuals contrast the apparent presence of pluripotent progenitors, as they described that most of the newly generated cells do not express neuronal markers (165, 191).

In rodents, the strongest indicator of adult cortical neurogenesis is the electron microscopic detection of newly generated [3H] thymidine cortical neurons in rodent brains (169). Later, they were further supported by the finding of proliferating progenitors in the murine cerebral cortex that were directed towards a neuronal fate (positive for DCX and/or the neuronal markers NeuN and Hu) (11) after a provoked insult in deep layers of the cerebral cortex.

These newly generated cells were able to establish connections with other regions, including the hypothalamus and spinal cord (192). Later, it was demonstrated that neurogenic progenitors included not only ectopic SVZ progenitors but also a local pool of cells ubiquitously residing in the adult rodent cerebral cortex, producing mainly glial cells but retaining the ability to produce new neurons (150, 193). Among the different layers of the cerebral cortex, layer I has been suggested to harbor the highest neurogenic potential, given its importance during development and in early postnatal weeks both in primates and in rodents (10, 194) (**Figure 3**). This neurogenic activity has been demonstrated in adulthood after ischemic insult, with new neurons being generated in layer I and integrated into inner cortical layers (195).

Cortical oligodendrogenesis has been assessed in other pathological contexts, including demyelinating diseases and especially MS. Its potential to repair demyelinating lesions has been investigated in murine models, and the existence of the process in the human MS context has also been demonstrated (50, 196) (**Figure 3**). In this context, it is of great importance to understand the mechanisms underlying this process, and again,THs arise as a key candidate to regulate these processes.

As was thoroughly reviewed by Wang and colleagues (197), MCT8 and OATP1C1 have been consistently detected in the cerebral cortex of different species, including rodents, non-human-primates and humans, through all stages of development. This study has for the first time precisely reported the cellular location of both transporters. Aside from the classically described expression in blood vessels, the authors have shown neuronal expression of both transporters, especially in layer I of the cerebral cortex (197) (**Figure 3**). In particular, OATP1C1 was strongly detected in pyramidal neurons. As DIO2 has not been demonstrated to colocalize in these neurons (138), the presence of the transporter could indicate either increased importance of T_4_ or, as the authors suggest, the ability of these neurons to accumulate T_4_ and release it when necessary to other surrounding cell types. Interestingly, the authors described the expression of OATP1C1 in Corpora amylacea. These globular structures, equally located in cortical layer I, have been described to also express the TH-distributor protein TTR (198), suggesting a function in the storage/buffering and/or delivery of T_4_ in layer I upon local need.

Despite the various lines of evidence on adult cortical neurogliogenesis and the control of TH availability occurring locally in different cortical areas, the role of THs in cortical neurogliogenesis remains to be clarified.

### Future challenges:

- Are THs a factor in cortical neurogliogenesis regulation?
- Is this regulation linked to parenchymal or cell-by-cell intracellular THs levels?
- Is this cell-by-cell regulation being carried out by progenitors present at the niches, as if the maintenance of neurogliogenic potential depends on paracrine-like TH signaling inside the niche?
- Would it be possible to manipulate TH availability or TH downstream effectors expression to regulate neurogliogenesis at the cellular level, to avoid the side effects derived from excessive TH signaling?

## Conclusion

Since the seminal discovery of neurogliogenic niches in the adult mammalian brain, enormous progress has been made in understanding the role of adult-generated neurons and glial cells in health and disease. In this review, we have highlighted THs as a key factor that controls neurogliogenic fate decisions and lineage progression at multiple levels. To that end, we have summarized existing knowledge on the precise spatiotemporal regulation of TH availability in progenitor and mature cells. Yet, many questions as to the cell-specific role of THs and TH signaling regulators as well as the functional outcome of altered TH availability are still unanswered. Future research employing new model systems and advanced methodology is needed to close those gaps. Likewise, past investigations have mainly focused on the “classical” neurogliogenic niches in the SVZ and SGZ, while the putative role of TH signaling in emerging niches in the hypothalamus, striatum, and cerebral cortex is still largely elusive. By pointing out critical open questions we aspired to spark future studies to fill in the blanks. The expected answers will help to fully harness THs’ potential to shift the fate of progenitor cells and thus foster regenerative processes in pathological conditions when new neurons and/or new oligodendrocytes are urgently needed.

## Author contributions

VV-H: Conceptualization, Writing – original draft, Writing – review & editing. SM: Conceptualization, Writing – original draft, Writing – review & editing. AG-F: Conceptualization, Writing – original draft, Writing – review & editing. SR: Conceptualization, Writing – original draft, Writing – review & editing.
